# The Functions of Cytochrome P450 ω-hydroxylases and the Associated Eicosanoids in Inflammation-Related Diseases

**DOI:** 10.3389/fphar.2021.716801

**Published:** 2021-09-14

**Authors:** Kai-Di Ni, Jun-Yan Liu

**Affiliations:** Center for Novel Target and Therapeutic Intervention, Institute of Life Sciences, Chongqing Medical University, Chongqing, China

**Keywords:** cytochrome P450, omega hydroxylase, eicosanoids, inflammation, cardiovascular disease

## Abstract

The cytochrome P450 (CYP) ω-hydroxylases are a subfamily of CYP enzymes. While CYPs are the main metabolic enzymes that mediate the oxidation reactions of many endogenous and exogenous compounds in the human body, CYP ω-hydroxylases mediate the metabolism of multiple fatty acids and their metabolites via the addition of a hydroxyl group to the ω- or (ω-1)-C atom of the substrates. The substrates of CYP ω-hydroxylases include but not limited to arachidonic acid, docosahexaenoic acid, eicosapentaenoic acid, epoxyeicosatrienoic acids, leukotrienes, and prostaglandins. The CYP ω-hydroxylases-mediated metabolites, such as 20-hyroxyleicosatrienoic acid (20-HETE), 19-HETE, 20-hydroxyl leukotriene B4 (20-OH-LTB_4_), and many ω-hydroxylated prostaglandins, have pleiotropic effects in inflammation and many inflammation-associated diseases. Here we reviewed the classification, tissue distribution of CYP ω-hydroxylases and the role of their hydroxylated metabolites in inflammation-associated diseases. We described up-regulation of CYP ω-hydroxylases may be a pathogenic mechanism of many inflammation-associated diseases and thus CYP ω-hydroxylases may be a therapeutic target for these diseases. CYP ω-hydroxylases-mediated eicosanods play important roles in inflammation as pro-inflammatory or anti-inflammatory mediators, participating in the process stimulated by cytokines and/or the process stimulating the production of multiple cytokines. However, most previous studies focused on 20-HETE,and further studies are needed for the function and mechanisms of other CYP ω-hydroxylases-mediated eicosanoids. We believe that our studies of CYP ω-hydroxylases and their associated eicosanoids will advance the translational and clinal use of CYP ω-hydroxylases inhibitors and activators in many diseases.

## Introduction

**FIGURE 1:**
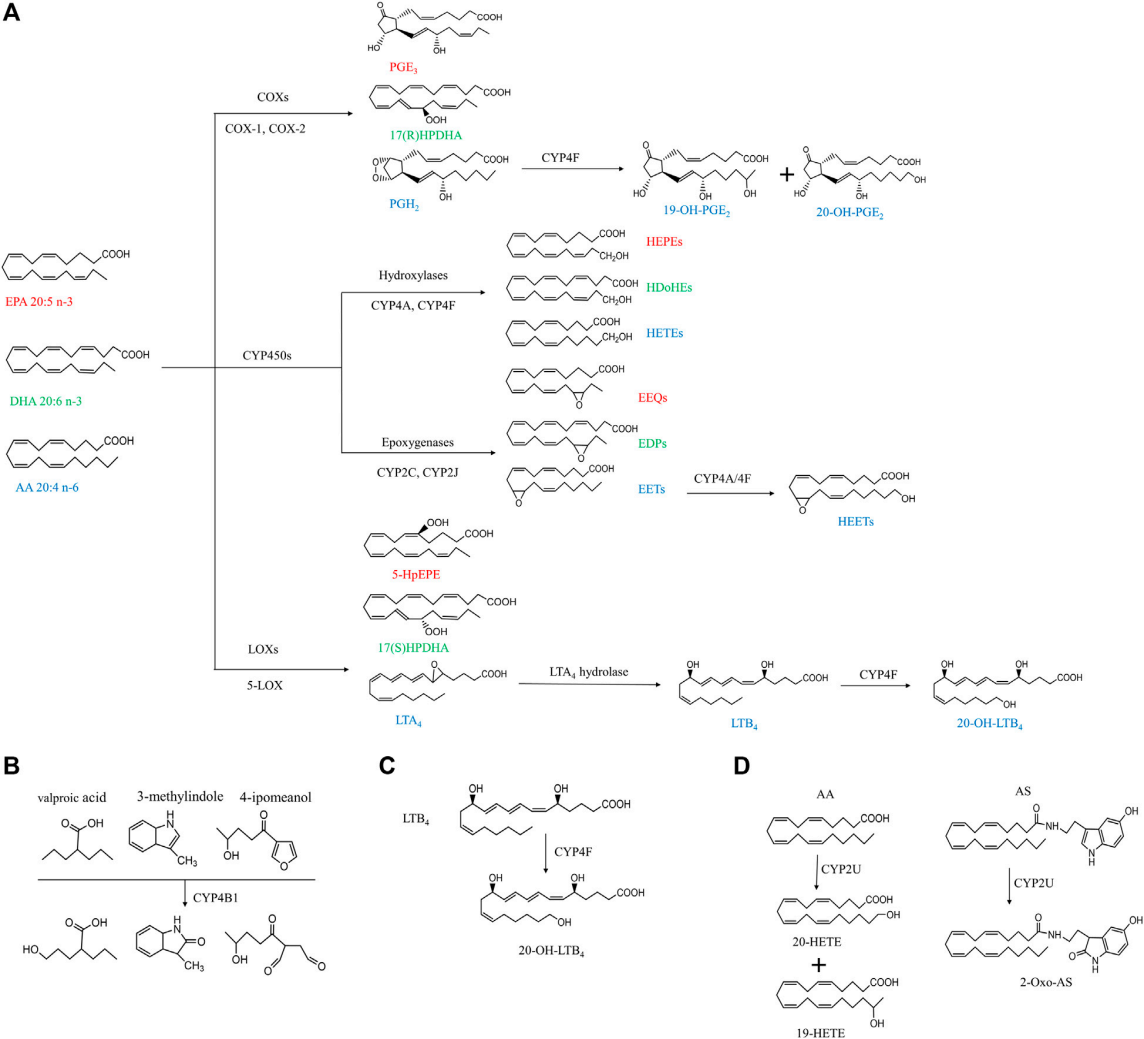
Simplified cascade of CYP ω-hydroxylases-mediated substrates and associated metabolites. The compounds with the same color indicate they are derived from the same substrates. AA, arachidonic acid; AS, *N*-arachidonoylserine; COX, cyclooxygenase; CYP, cytochrome P450; DHA, docosahexaenoic acid; EET, epoxyeicosatrienoic acid; EEQ, epoxyeicosatetreaenoic acid; EDPs, epoxydocosapentaenoic acid; EPA, eicosapentaenoic acid; HEPEs, hydroxyeicosapentaenoic acid; HEETs, hydroxyepoxyeicosatrienoic acids; HPDHA, hydroperoxydocosahexaenoic acid; HpEPE, hydroperoxyeicosapentaenoic acid; HETE, hydroxyeicosatetraenoic acid; HDoHE, hydroxydocosahexaenoic acid; LOX, lipoxygenase; LTA_4_, leukotriene A_4_; LTB_4,_ leukotriene B_4;_ PGH_2_, prostaglandin H_2_; PGE_3_, prostaglandin E_3_.

Cytochrome P450 (CYP) enzymes, discovered in the early 1960s, are a superfamily of heme containing membrane bound monoxygenases which is available in microorganisms, plants, animals, and humans (Guengerich et al., 2016; Elfaki et al., 2018). About 300,000 CYP sequences have been collected from public and private sources (Nelson, 2018). The common reactions catalyzed by CYPs include hydroxylation, heteroatom oxygenation and release, epoxidation, and oxidation of double, triple, or aromatic π-bonds (Guengerich, 2001; McIntosh et al., 2014; Ortiz de Montellano, 2019). Mammalian CYP enzymes are distributed in a variety of tissues and organs of organisms, and play a core role in cell metabolism to maintain cell homeostasis mainly by mediating the metabolism of a large number of xenobiotic and endobiotic molecules, including but not limited to drugs, industrial toxins, steroids, cholic acid, and fatty acids through regio-, chemo- and stereospecific oxidation, peroxidation and reduction (Urlacher and Girhard, 2012; Manikandan and Nagini, 2018). There are 57 CYP genes and 58 pseudogenes in human and are divided into 18 families and 43 subfamilies (Waring, 2020), which are mainly present in the kidney, small intestine and liver tissues (Elfaki et al., 2018). The CYP ω-hydroxylases, are a group of subfamilies of CYPs that mediate the metabolism of multiple fatty acids via the addition of a hydroxyl group to the ω- or (ω-1)-C atom of the substrates. This includes polyunsaturated fatty acids (PUFAs), such as arachidonic acid (AA), eicosapentaenoic acid (EPA), docosahexaenoic acid (DHA) and their derivatives (Figure 1). Those metabolites derived from AA, EPA and DHA are the members of eicosanoids and function as inflammatory mediators, which play an important role in the occurrence and progression of many pathological conditions like cardiovascular disease, cancer and diabetes (Westphal et al., 2011; Schunck et al., 2018; Colombero et al., 2020). This article reviews the activity and expression changes of CYP ω-hydroxylase in inflammation-related diseases, and the enzyme-mediated metabolites, such as 20-HETE, which trigger the downstream signaling pathway and induce more pathological changes.

## Metabolism of n-3 and n-6 PUFAs

As shown in Figure 1, PUFAs can undergo three main enzymatic pathways: cyclooxygenase (COXs), CYP and lipoxygenase (LOXs). COXs convert EPA, DHA and AA into PGH2, PGE3, prostaglandin E3 (PGE3), 17(R)-hydroperoxydocosahexaenoic acid (17(R)HPDHA) and prostaglandin H2 (PGH2) (Smith and Song, 2002; Johnson et al., 2015). PGH2 can be further hydroxylated by CYP4F8 to 19-OH-PGH2. Human have six different kinds of LOXs (5-LOX, 12-LOX, 12/15-LOX, 15-LOX type 2, 12(R)-LOX, and epidermal LOX), and 5-LOX is a key enzyme in leukotriene biosynthesis in health and disease (Rådmark et al., 2015). EPA, DHA and AA can be metabolized by 5-LOX to 5-hydroperoxyeicosapentaenoic acid (5-HpEPE), 17(S)HPDHA and leukotriene A4 (LTA4), respectively. The metabolism by CYP pathways has been described in detail in our previous review and will not be included here (Luo and Liu, 2020).

## Classification, Tissue Distribution, and Biological Characteristics of Cytochrome P450 omega hydroxylases

The human CYP enzymes that catalyze ω-hydroxylation of fatty acids include CYP4A, CYP4B, CYP4F, and CYP2U1 (Chuang et al., 2004; Hardwick, 2008) (Table1). These CYP enzymes can hydroxylate saturated fatty acids, branched fatty acids, unsaturated fatty acids, and some eicosanoids (Figure 1).

**TABLE 1:** CYP ω-hydroxylase orthologous genes expressed in various mouse and human organs.

Human	Organs	Cell type specificity	Mouse	Organs	Cell type specificity	References
***4A11***	liver, kidney, small intestine, lung, heart, skin, adrenal, prostate, testis, uterus, mammary, placenta	hepatocytes, proximal tubular cells	*4a10*	liver, kidney		Hrycay and Bandiera, (2009)
***4A22***	liver, kidney	hepatocytes	*4a12a*	liver, kidney	smooth muscle cell embryonic fibroblast	Hrycay and Bandiera. (2009); Yue et al. (2014)
			*4a12b*	liver, kidney, lung		
			*4a14*	liver, kidney		
			*4a29*	testis, thmus		
			*4a30b*	colon, testis		
			*4a31*	kidney, liver		
			*4a32*	kidney, liver		
***4B1***	small intestine, lung, kidney, heart, skin, spleen, thymus, pancreas, skeletal muscle, eye, adrenal, prostate, urinary bladder, testis, uterus, mammary, placenta	alveolar cells type 1, ciliated cells, club cells	*4b1*	liver, kidney, lung, brain, skeletal muscle, spleen, testis, small intestine		Hrycay and Bandiera. (2009); Yue et al. (2014)
***4F2***	liver, small intestine, kidney, brain, skin, prostate, testis	hepatocytes	*4f13*	liver, kidney, lung, heart, testis		Choudhary et al. (2003); Hrycay and Bandiera. (2009)
***4F3***	liver, small intestine, trachea, kidney, prostate	hepatocytes	*4f14*	liver, kidney, brain, testis		Hrycay and Bandiera, (2009)
***4F8***	small intestine, lung, stomach, kidney, skin, eye, adrenal, prostate, urinary bladder, testis, uterus.	urothelial cells, glandular cells, granulocytes	*4f15*	liver, kidney, lung, brain	astrocyte, mesodermal cells	Hrycay and Bandiera, (2009)
***4F11***	liver, colon, heart, brain, skeletal muscle, ovary, placenta, kidney	hepatocytes, ductal cells, urothelial cells	*4f16*	liver, kidney, lung, brain, heart, spleen		Hrycay and Bandiera, (2009)
***4F12***	liver, small intestine, stomach, colon, kidney, heart, skin, prostate, ovary, placenta	enterocytes, paneth cells, undifferentiated cells	*4f17*	ubiquitous expression in subcutaneous fat pad adult, ovary and 26 other tissues	smooth muscle cells, cardiomyocytes, astrocyte	Hrycay and Bandiera, (2009)
***4F22***	liver, small intestine, kidney, brain, skin, skeletal muscle, testis, placenta	granulocytes, Suprabasal	*4f18*	liver, kidney, lung, spleen, ovary		Hrycay and Bandiera. (2009); Yue et al. (2014)
		keratinocytes, glandular cells	*4f37*	duodenum, large intestine and 20 other tissues	skin langerhans cells, spermatid, dendritic cells	
		spermatogonia	*4f39*	stomach, testis, bladder, kidney, lung	spermatid	
			*4f40*	testis, colon, duodenum	embryonic stem cells	
***4V2***	eye, ovary	hepatocytes, muller glia cells	*4v3*	liver, eye		Hrycay and Bandiera, (2009)
***4X1***	liver, small intestine, trachea, lung, colon, kidney, heart, brain, skin, spleen, thymus, pancreas, skeletal muscle, prostate, testis, ovary, uterus, mammary, placenta	ciliated cells	*4x1*	liver, kidney, lung, brain, heart, spleen	Neuron	Hrycay and Bandiera, (2009)
***4Z1***	liver, kidney, skeletal muscle, mammary, ovary	alveolar cells type 1	*None*			Hrycay and Bandiera, (2009)
***2U1***	thymus, heart, brain, bladder, prostate, uterus, testis, kidney, liver, lung, spleen, skeletal muscle, trachea, salivary gland, skin, pancreas, adrenal	melanocytes, peritubular cells	*2u1*	thymus, brain, heart, liver, testis, kidney, lung, skeletal muscle, spleen, skin		Chuang et al. (2004); Karlgren et al. (2004); Choudhary et al. (2005)

The CYP4A subfamilies are found in mammals, including human, rat, and mice, and are mainly expressed in the liver and kidney (Simpson, 1997). The mouse *Cyp4a* subfamily includes *Cyp4a10*, *Cyp4a1a*, *Cyp4a12b*, and *Cyp4a14*. In mice, *Cyp4a* mRNA expression levels in the liver and kidney are regulated by sex hormones and/or growth hormones (Zhang and Klaassen, 2013). In human, there are two highly homologous CYP4A genes (*CYP4A11* and *CYP4A22*) located on chromosome 1, and showed 96% sequence identity (Bellamine et al., 2003; Savas et al., 2003; Hsu et al., 2007). However, rat CYP4 has four members (genes *Cyp4a1*, *Cyp4a2*, *Cyp4a3*, and *Cyp4a8*). CYP4A subfamily proteins metabolize arachidonic acid to produce 19-hydroxyeicosatetraenoic acid (19-HETE) and 20-HETE, playing an important role in lipid homeostasis related to fatty acids and eicosanoic acids. Several studies have shown that CYP4A11 contributes about 13 and 33% to the formation of 20-HETE by ω-hydroxylation of arachidonic acid in the human liver and kidney (Powell et al., 1998; Lasker et al., 2000). The functions of the CYP4A22 have not been elucidated fully.

The tissue distribution of CYP4B1 varies widely among species. *Cyp4b1* was originally discovered from the rabbit lung in the mid-1970s (Arinç and Philpot, 1976). In mice, *Cyp4b1* expression is predominantly present in the brain, lung, and small intestine, while low in the spleen, testis, liver, and skeletal muscle (Baer and Rettie, 2006). Human CYP4B1 is mainly found in lung microsomes, accounting for 70% of the total, and remaining parts in the heart, skeletal muscle, kidneys, and prostate glands (Choudhary et al., 2005). CYP4B1 is specialized in the ω-hydroxylation of short-chain fatty acids and the metabolism of exogenous compounds including valproic acid, 3-methylindole, 4-ipomeanol, 3-methoxy-4-aminoazobenzene, and many aromatic amines (Figure 1B) (Baer and Rettie, 2006). The tissue specificity, genetic polymorphisms, and metabolic capabilities of human CYP4B1 are still under investigation because of the difficulty in allogeneic expression of the human *CYP4B1* gene.

Human has seven CYP4F enzymes encoded by six different genes in the *CYP4F* gene cluster (19p13.1) on chromosome 19. CYP4F2 enzyme, also known as leukotriene B_4_ (LTB_4_) omega-hydroxylase, is located on chromosome 19 p13.11. CYP4F2 is approximately 20 kbp, consisting of 13 exons and 12 introns encoding 520 amino acids (Kikuta et al., 1999). It is mainly distributed in tissues and organs such as liver, kidney, lung, white blood cells, and particularly endoplasmic reticulum (Hsu et al., 2007; Hirani et al., 2008). CYP4F2 is a monooxygenase that catalyzes many reactions, including drug metabolism, the synthesis and metabolism of lipids, steroids, and cholesterol. It can affect the metabolism of AA and catalyze LTB_4._, a metabolite of AA mediated by (5-LOX), serving as the main ω-hydroxylase of AA and LTB_4_. Eun et al. found that the mRNA expression levels of *CYP4F2* and *CYP4F12* in hepatocellular carcinoma tissues were significantly lower than those in normal liver tissues, which was closely related to the overall survival rate of patients with hepatocellular carcinoma (Eun et al., 2018). *CYP4F3*, an unusual human CYP gene, was initially identified as the ω-oxidase that catalyzes LTB_4_ in human neutrophils (Shak and Goldstein, 1984; Kikuta et al., 1993; Christmas et al., 2001). Christmas *et al.* subsequently identified an alternative splice form of CYP4F3 in the liver and specified two subtypes, CYP4F3A and CYP4F3B (Christmas et al., 2001). CYP4F3A is expressed in neutrophils but not in the liver and has a very high affinity to LTB_4_. In contrast, CYP4F3B is mainly expressed in the human liver and kidney, but not in myeloid cells, which is more active in the hydroxylation of AA and other ω-3 polyunsaturated fatty acids (PUFA) than in hydroxylating LTB_4_ (Fer et al., 2008). In rat, Cyp4f6 converts LTB_4_ to form 19- and 18-hydroxy-LTB_4_ with an apparent K(m) of 26 M and Cyp4f5 converts LTB_4_ predominantly to 18-hydroxy-LTB_4_ with an apparent K(m) of 9.7 M (Figure 1C). CYP4F5 and CYP4F6 are active in the lung and to some extent in the brain, kidney and testis. CYP4F5 and CYP4F6, due to their ability to metabolize LTB_4_, may play an important role in regulating the inflammatory response in these organs (Bylund et al., 2003).

Human CYP4V2 protein is expressed in eye, ovary, and liver (Li et al., 2004), while mouse Cyp4v3 is mainly detected in the liver and retina (Jenkins et al., 2006; Liu et al., 2006). Human *CYP4X1* is very widely expressed transcriptionally in adult human tissues, predominantly in skeletal muscle, trachea, and aorta (Hsu et al., 2007). Al-Anizy et al. reported that Cyp4x1 was a major CYP protein in mouse brain (Al-Anizy et al., 2006). *CYP4Z1* gene is a unique CYP4 gene in human, and no orthologous gene has been found in mice at present. CYP4Z1 is mainly distributed in human liver, kidney, skeletal muscle, testis, and mammary, and is highly expressed in breast cancer, and a regulator of tumor angiogenesis and growth of breast cancer (Yu et al., 2012; Wang et al., 2016; Nunna et al., 2017; Yang et al., 2017). The substrates of CYP4Z1 and associated metabolism have not been fully understood.

CYP2U1 is an “orphan” enzyme which was originally identified as a member of CYP2 subfamily by Chuang et al. (2004) and Karlgren et al. (2004). To date, the *CYP2U1* gene, the only reported member of the CYP2U subfamily, is over 18 kb long and located on chromosome 4q25 (Devos et al., 2010). Human CYP2U1 shares 89 and 83% amino acid sequence identity with rat and mouse Cyp2u1, respectively (Dhers et al., 2017). Studies have shown that human *CYP2U1* mRNA is expressed predominantly in thymus and cerebellum, and similar findings were observed in rat and mice (Chuang et al., 2004; Karlgren et al., 2004; Dhers et al., 2017). However, human CYP2U1 protein was only detected in brain, platelets and megakaryocytic Dami cells (Dhers et al., 2017). Likewise, in rat Cyp2u1 protein was also present only in the cerebellum and thymus (Karlgren et al., 2004). CYP2U1 showed hydroxylase activity for fatty acids and *N*-arachidonoylserine (AS) (Figure 1D) (Dhers et al., 2017). Although CYP2U1 has been shown to be involved in some diseases such as breast cancer and hereditary spastic paraplegia, the biological role is still largely unknown (Luo et al., 2020). The cell-specific distribution of CYP ω-hydrolases is key to the local pro-inflammatory effects observed across various diseases. While a systemic study of the cell-specific distribution of these enzymes was lacked, CYP4A, CYP4F, and CYP4B1 have been frequently investigated in epithelial cells, endothelial cells, platelet and immunocytes (Table 1) (Kikuta et al., 2002; Kikuta et al., 2004; Cheng et al., 2014; Li et al., 2015; Chen et al., 2019).

## Orthologous Cytochrome P450 ω-hydroxylase Genes in Human and Mice

Many different species share homology of genes. Generally, two genes are homologous genes when their sequence similarities are over 80%. Homologous sequences can be further divided into two types: orthology and paralogy (Koonin, 2005). A recent study showed that 84% of mouse-human orthologous genes have been conservatively evolved in the expression profiles (Hrycay and Bandiera, 2009). Thirty six pairs of orthologous CYP genes have been found to perform similar or identical functions in human and mice, which facilitates to study the functions of human CYPs by using murine models (Nelson et al., 2004). The CYP ω-hydroxylase orthologous genes in human and mice are shown in Table1.

## Effects of Gender on Cytochrome P450 ω-hydroxylase

The expression of CYP ω-hydroxylases has gender differences. Cyp4a10 is expressed in both male and female mice, while Cyp4a12a is male-specific and regulated by androgen, and Cyp4a14 is strongly expressed in female mice (Wu et al., 2013). Cyp4a14 (−/−) mice have been found to exhibit male-specific hypertension. Whereas administration of androgens to male or female rat or mice results in hypertension (Holla et al., 2001). Both 20-HETE and androgens have been found to be strongly associated with hypertension and other cardiovascular diseases (Reckelhoff, 2005; Ward et al., 2005). However, the connection and potential mechanism between Both 20-HETE and androgens have not been clarified.

## Cytochrome P450 ω-hydroxylases and Inflammation

CYP4A, CYP4B, CYP4F, and CYP2U1 are the subfamilies of CYP ω-hydroxylases that catalyze the hydroxylation of AA, other medium- and long-chain fatty acids, and the derivatives of fatty acids like LTB_4,_ EETs, and prostaglandins. The CYP ω-hydroxylases-mediated metabolites derived from above-mentioned substrates, particularly 20-HETE, have been shown to play a vital role in inflammatory diseases. Here, we discuss the role of CYP ω-hydroxylases in inflammation.

Recent studies have shown that inflammation could significantly decrease the expression of CYP monooxygenases in the heart, kidney, and liver, while increase the expression of CYP ω-hydroxylases. As a result, CYP ω-hydroxylase mediated conversion of the corresponding metabolites of EETs were decreased, while 20-HETE was increased. These changes may participate in the onset and progression of various diseases through inflammatory response (Anwar-mohamed et al., 2010). In an *in-vivo* study, salidroside can facilitate reprogramming of CYP4A-mediated arachidonic acid metabolism in macrophages in the treatment of monosodium urate crystal-induced gouty arthritis. The study reported that salidroside could reduce the production of inflammatory factors TNF-α and IL-1β by down-regulating CYP4A to polarize macrophages away from the M1 phenotype, and ameliorate inflammation (Liu et al., 2019). Ashkar *et al.* found that retinoic acid induces corneal epithelial *CYP4B1* gene expression and stimulates the synthesis of inflammatory 12-hydroxyeicosanoic acid (Ashkar et al., 2004).

In a rodent model of lipopolysaccharide (LPS)-induced inflammatory infection and injury, the mRNA expressions of *Cyp4f4* and *Cyp4f5* were decreased by 50 and 40%, respectively, in the liver, while the concentrations of leukotrienes and prostaglandins were increased. When *Cyp4f* was up-regulated, leukotrienes and prostaglandin mediators were decreased, thus alleviating inflammation (Cui et al., 2003). The decrease in leukotrienes and prostaglandins caused by upregulation of Cyp4f may be accounted for the metabolic shunting among CYPs, COXs, and LOXs, and/or Cyp4f-mediated metabolism of leukotrienes and prostaglandins. In addition, Kalsotra *et al.* reported that in a rat model of traumatic brain injury, inflammatory cells in the airway and alveolar space migrated extensively, and further secondary damage could be relieved by reducing LTB_4_ via activating LTB_4_ decomposition by induced CYP4Fs, which opened up new possibilities for the treatment of post-traumatic pulmonary inflammation (Kalsotra et al., 2007b). CYP4F2, the major LTB_4_ hydroxylase expressed in human liver, may play an important role in regulating the circulation and liver levels of LTB_4_ (Johnson et al., 2015). In addition to LTB_4_, it was also found that lipoxin A4 (LXA_4_) and hydroxyeicosanoic acid in rodent hepatocytes could be degraded via the ω-hydroxylation by recombinant CYP4Fs. Proinflammatory cytokines, such as IL-1β, IL-6, and TNF-α, induce CYP4Fs via STAT3 signaling. The anti-inflammatory factor IL-10 inhibits the expression of CYP4F (Kalsotra et al., 2007a).

With the continuous innovation and development of biotechnology, research tools of chemical synthesis and gene editing continue to expand, research efficiency of CYP ω-hydroxylase is greatly improved. The associations of CYP ω-hydroxylases with pathogenesis of diseases are gradually discovered. Currently, activators and inhibitors of CYP ω-hydroxylase isomers, and CYP ω-hydroxylase knockout (KO) and transgenic mice are gradually being utilized in many studies. Tables 2, 3 summarizes the commonly used inhibitors and inducers of CYP ω-hydroxylase and CYP ω-hydroxylase KO and transgenic mice models.

**TABLE 2:** The inhibitors and inducers of CYP ω-hydroxylase.

Drug	Inhibitor/Inducer	References
N-hydroxy-N'-(4-butyl-2-methylphenyl)-formamidine (HET0016)	selective inhibitor of 4A	Sato et al. (2001); Guo et al. (2005)
12,12-dibromododec-11-enoic acid (DBDD)	selective inhibitor of 20-HETE synthesis	Kroetz and Xu, (2005)
10-undecynyl sulfate (10-SUYS)	selective inhibitor of 20-HETE synthesis	Kroetz and Xu, (2005)
N-methylsulfonyl-12,12-dibromododec-11-enamide (DDMS)	selective inhibitor of 20-HETE synthesis	Kroetz and Xu, (2005)
TS-011	selective inhibitor of 20-HETE synthesis	Miyata et al. (2005); Edson and Rettie, (2013)
Flavonoid (FLA-16)	selective inhibitor of 4A	Wang et al. (2017)
Terminal acetylenic fatty acids (17- ODYA)	nonselective inhibitor	Kroetz and Xu, (2005)
1-aminobenzotriazole (ABT)	nonselective inhibitor	Kroetz and Xu, (2005); Sun et al. (2011)
Acetylshikonin	nonselective inhibitor	Shon et al. (2017)
Fibrates	inducer of 4A11	Edson and Rettie, (2013)
Rifampicin	inducer of 4F12	Hariparsad et al. (2009)
Lovastatin	inducer of 4F2	Edson and Rettie, (2013)
Mevastatin	inducer of 4F2	Edson and Rettie, (2013)
Genistein	inducer of 4F2	Hsu et al. (2011); Edson and Rettie, (2013)
AICAR	inducer of 4F2	Bumpus and Johnson, (2011)
Resveratrol	inducer of 4F2	Edson and Rettie, (2013)

**TABLE 3:** CYP ω-hydroxylase KO and transgenic mouse model^a^.

Gene	Strain name	References
*Cyp4b1*	C57BL/6N -*Cyp4b1* ^tm1a(KOMP)Wtsi^	Baldarelli et al. (2021)
	C57BL/6N-Cyp4b1^tm1b(KOMP)Wtsi^	
*Cyp4f13*	Cyp4f13^Gt(OST14770)Lex^	
*Cyp4f14*	C57BL/6N-Cyp4f14^tm1a(EUCOMM)Hmgu/Ieg^	
	C57BL/6N-Cyp4f14^tm1b(EUCOMM)Hmgu/Ieg^	
*Cyp4f16*	C57BL/6N-Cyp4f16^tm1a(EUCOMM)Wtsi/BayMmucd^	
	C57BL/6N-Cyp4f16^tm1b(EUCOMM)Hmgu/BayMmucd^	
	C57BL/6N-Cyp4f16^tm1b(KOMP)Wtsi/H^	
	C57BL/6NTac-Cyp4f16^tm1a(KOMP)Wtsi/H^	
*Cyp4f18*	B6.Cg-Cyp4f18^tm1.1Pchr^	
	B6.Cg-Cyp4f18^tm1.2Pchr^	
	B6.Cg-Cyp4f18^tm1.1Pchr/Mmmh^	
	C57BL/6N-Cyp4f18^em1(IMPC)Wtsi/WtsiCnrm^	
*Cyp4v3*	B6(Cg)-Cyp4v3^tm1(KOMP)Vlcg^	
	C57BL/6NCrl-Cyp4v3^em1(IMPC)Mbp^/Mmucd	
*Cyp4x1*	B6;129S5-Cyp4x1tm1Lex/Mmucd	
	B6;129S5-Cyp4x1^tm1Lex^/Tac	
	C57BL/6-Cyp4x1^tm1Beld^/H	
*Cyp2u1*	B6N(Cg)-Cyp2u1^tm1b(EUCOMM)Wtsi^/J	
	B6N(Cg)-Cyp2u1^tm1a(EUCOMM)Wtsi^/J	
	C57BL/6N-A^tm1Brd^ Cyp2u1^tm1a(EUCOMM)Wtsi^/IcsOrl	
	C57BL/6N-A^tm1Brd^ Cyp2u1^tm1a(EUCOMM)Wtsi^/ JMmucd	
	C57BL/6N-A^tm1Brd^ Cyp2u1^tm1b(EUCOMM)Wtsi^/ JMmucd	

_a_ The information was collected from http://www.informatics.jax.org/.

## The Roles of Cytochrome P450 ω-Hydroxylase-mediated Eicosanoids in Inflammation-Associated Diseases

Eiconanoids have different modulating inflammation effects on cardiovascular system, brain, liver, and lung during pathological condition. Here, we summarized the effects of eiconanoids on inflammatory diseases in different tissues (Table 4). When these organs are damaged by inflammation caused by a variety of pathogenic factors, excessive inflammatory mediators including eicosanoids will be released locally, which can mediate the inflammatory reactions in local tissues (Wallace, 2019; Yao and Narumiya, 2019; Calder, 2020).

**TABLE 4:** Eicosanoids roles in inflammatory diseases.

Eicosanoids	Tissue	Effects	Disease	References
20-HETE	cardiovascular system	stimulation of smooth muscle cell contractility, migration, proliferation activation of endothelial cell dysfunction and inflammation	Hypertension, cardiac hypertrophy and myocardial infarction	Fan et al. (2016)
	kidney	inhibits sodium transport, blocks Na/K-ATPase and potassium channels, interacts with ANG II, dopamine, endothelin, and parathyroid hormone	Polycystic kidney disease, acute renal failure (AKI), and chronic kidney disease (CKD)	Imig, (2013); Fan and Roman, (2017)
	brain	regulates cerebral vascular tone	Stroke, subarachnoid hemorrhage (SAH)	Elshenawy et al. (2017)
	lung	contributes to the regulation of airway resistance and pulmonary vascular tone	obstructive airway diseases and asthma	Fan et al. (2016); Elshenawy et al. (2017)
19(S)-HETE	cardiovascular system	protects against angiotensin II (Ang II)-induced cardiac hypertrophy	Cardiac Hypertrophy	Elkhatali et al. (2015)
20-OH-LBT_4_	bronchus	unknown	nonallergic asthma	Bruijnzeel et al. (1993)

20-HETE is the major metabolite of arachidonic acid mediated by CYP ω-hydroxylase, which plays an important role in the regulation of cardiovascular disease, renal function disorder, carcinogenic condition, and other inflammatory diseases. CYP4A11 and CYP4F2 are the primary enzymes that mediate the formation of 20-HETE in human liver and kidney microsomes (Lasker et al., 2000). Vascular inflammation plays an important role in the occurrence of many diseases, including atherosclerosis, hypertension, and vascular remodeling. 20-HETE can promote vascular inflammation by increasing adhesion molecules and inflammatory cytokines due to endothelial cell activation (Hoopes et al., 2015). 20-HETE can activate nuclear factor-kappa B (NF-κB) and stimulate the production of inflammatory cytokines in human endothelial cells (Ishizuka et al., 2008). Recent studies have proved that 20-HETE could bind to the G-protein coupled receptor 75 (GPR75) to promote c-Src-mediated-EGFR and trigger the downstream MAPK pathway to induce ACE expression and endothelial dysfunction in human endothelial cells (Garcia et al., 2017; Pascale et al., 2021). 20-HETE/GPR75 also triggered PI3K/AKT pathway to promote vascular smooth muscle cells migration, hypertrophy. Moreover, 20-HETE/GPR75 is involved in the activation of intracellular signaling in prostate cancer cells, leading to the more aggressive phenotypic differentiation of PC-3 cells (Cárdenas et al., 2020). In endothelial cells, 20-HETE can promote reactive oxygen species (ROS) production through NADPH oxidase to activate the L-type Ca^2+^channel (Medhora et al., 2008; Zeng et al., 2010; Bou-Fakhredin et al., 2021). In the ischemia-reperfusion injury, inhibition of 20-HETE synthesis reduced oxidative stress and the expression of vascular TNFα, IL-1β and IL-6 (Regner et al., 2009; Hoff et al., 2011). In addition, Han *et al.* found that the use of 20-HETE synthesis inhibitor HET0016 to inhibit the synthesis of 20-HETE can reduce the volume of brain injury and neurological deficit, alleviating neuronal death, ROS production, gelation activity, and inflammatory reaction, which indicates that inhibition of 20-HETE synthesis protects brain injury after intracerebral hemorrhage without inhibiting angiogenesis (Han et al., 2019; Cui et al., 2021). Inhibition of 20-HETE production can also attenuate kidney injury in a rodent model of acute kidney injury (AKI) induced by ischemia/reperfusion (I/R) (Hoff et al., 2011; Hoff et al., 2019). 20-HETE promotes tumor angiogenesis and metastasis by upregulation of VEGF and MMP-9 via PI_3_K and ERK1/2 signaling in the human NSCLC cells (Yu et al., 2011). Increased expression of CYP4A and CYP4F enzymes in human cancer tissues and the use of 20-HETE inhibitors and antagonists in the treatment of cancer have been reported (Amet et al., 1998).

In humans, 19-HETE is mainly synthesized by the CYP2C19 and CYP2E1 pathways, with less synthesis by the CYP ω-hydroxylase pathway (Shoieb et al., 2019). In normal physiology, 19-HETE can function as an endogenous antagonist of 20-HETE in mediating renal vasoconstriction by blocking the vasoconstriction of renal arterioles caused by 20-HETE (Shoieb et al., 2019). It has been reported that CYP-mediated 19-HETE has a strong correlation with cardiovascular events and can act as a prognostic marker for patients with acute coronary syndrome (Shoieb et al., 2019). It should be noted that 19-HETE was usually investigated as a racemic mixture, however, 19(S)-HETE was reported more active than 19(R)-HETE against Ang II-cell hypertrophy (Shoieb and El-Kadi, 2018). In the heart, 19-HETE is the major subterminal HETE formed in the cardiac tissue of rat, which not only plays a protective role in cardiac hypertrophy, but also participates in the pathogenesis of chronic kidney diseases (Kajiwara et al., 2013; El-Sherbeni and El-Kadi, 2014; Shoieb et al., 2019).

## Cytochrome P450 ω-Hydroxylase-mediated Products of LTB_4_

LTB_4_ is an inflammatory mediator involved in inflammatory diseases such as rheumatoid arthritis, asthma and Alzheimer’s disease, which can be metabolized by CYP4F2, CYP4F3A and CYP4F3B to form 20-OH-LTB_4_ (Kalsotra and Strobel, 2006) (Lorenzetti et al., 2019) (Brain and Williams, 1990; Wang et al., 2008). LTB4 is converted by CYP4F to the more polar 20-OH-LTB_4_ in human polymorphonuclear leukocytes (PMN) (Soberman et al., 1988). However, 20-OH-LTB_4_ expressed similar functional activity to LTB_4_, and similar binding characteristics with human PMN to LTB_4_. This indicated that the arachidonic acid metabolite oxidized at ω-site of LTB_4_ may be a more important inflammatory factor than LTB_4_ (Clancy et al., 1984). Analysis of peritoneal metabolites in patients with purulent peritonitis or non-performatives appendicitis revealed that 20-OH-LTB_4_ might be involved in the pathophysiological mechanisms of suppurative inflammation (Kikawa et al., 1986). A recent study showed that 20-OH-LTB_4_ might function as a potential biomarker for the diagnosis and risk assessment of intracerebral hemorrhage stroke (ICH) to distinguish the patients with ICH from healthy people and the patients with acute ischemic stroke (AIS). This finding provides a new strategy for the diagnosis, prevention and treatment of ICH (Zhang et al., 2021). In mouse myeloid cells, Cyp4f18 (the functional orthologue of human PMN CYP4F3A) catalyzes the conversion of LTB_4_ to 19-OH-LTB_4_. Inhibition of Cyp4f18 led to a 220% increase in the PMN chemotaxis to LTB_4_ in mice (Christmas et al., 2006). While the ω-hydroxylated products of LTB_4_ play different physiological roles in some diseases, the mechanisms in inflammation are still unclear, which needs further study.

## Cytochrome P450 ω-Hydroxylase-mediated Products of Epoxyeicosatrienoic Acid

*In vivo*, EETs are not only hydrolyzed by sEH and mEH, but also metabolized by CYP ω-hydroxylases. EETs are one of the best endogenous substrates for rat Cyp4a subtypes so far. 8(9)-, 11(12)- and 14(15)-EET could be metabolized by rat Cyp4a into corresponding 19- and 20-hydroxylated EET (HEET) (Cowart et al., 2002). Cyp4a1 showed a higher affinity for 8(9)-EET, while Cyp4a2, Cyp4a3, and Cyp4a8 have a higher hydroxylase activity for 11 (12)-EET (Cowart et al., 2002). ω-HEETs could also serve as endogenous PPARα ligands (Muller et al., 2004). Muller *et al.* reported that CYP-dependent production of EET/HEET might be an anti-inflammatory index (Muller et al., 2004). However, there is no evidence to show the functions of ω-hydroxylation of EET in humans (Xu et al., 2011).

## Cytochrome P450 ω-Hydroxylase-mediated Products of Prostaglandins

Since 1971, a series of studies have identified the Cyp4a hydroxylase family from multiple organs in rabbit and mouse liver (Kikuta et al., 2002). These enzymes catalyze the hydroxylation of multiple prostaglandins (PGE_1_, PGE_2_, PGF_2_, PGD_2_, PGA_1_, and PGA_2_) as well as ω- and (ω-1)-hydroxylation of palmitate. In humans, CYP4A11 can hydroxylate three PGH_2_ analogs (U51605, U44069, U46619), although it cannot hydroxylate PGH_2_ (Oliw et al., 2001). Moreover, PGH_2_ could be converted by CYP4F8 into 19(R)-OH-PGH_2_ in prostate, seminal vesicles, and several extrahepatic tissues (Oliw et al., 1988; Hardwick, 2008). PGE_2_ is closely related to the production of cytokines in antigen presenting cells and plays an important role in the stage of inflammatory regression, while 19(R)-OH-PGE_2_ is an agonist of PGE_2_ receptor (Serhan et al., 2007). At present, PGs have been studied extensively but little is known about the function of their hydroxylated products, and further studies are required to determine the function in various tissues and species.

## Cytochrome P450 ω-Hydroxylase-mediated Eicosanoids and Cytokines

CYP ω-hydroxylase-mediated eicosanoids are also involved in the regulation of cytokines, especially 20-HETE in cardiovascular inflammation has been widely studied. Cheng et al. found that 20-HETE could mediate the endothelial nitric oxide synthase (eNOS) uncoupling and endothelial dysfunction through activating tyrosine kinase, MAPK and IKK in bovine aortic endothelial cells (Cheng et al., 2010). In addition, 20-HETE can also stimulate NF-κB and MAPK/ERK to increase protein expression levels of IL-8 and adhesion molecule ICAM, leading to endothelial cell activation (Ishizuka et al., 2008; Cheng et al., 2010). In the spontaneously hypertensive rat model, the inhibition of 20-HETE by HET006 (CYP ω-hydroxylase inhibitor) could significantly reduce oxidative stress and the mRNA expression of TNFα and IL-1β, and the NF-κB activation (Toth et al., 2013). Cheng et al. developed a new constitutively stimulated 20-HETE biosynthesis mouse model, the Tie2-CYP4F2-Tr mouse. By activating the NADPH oxidase and VEGF pathway, the model has the phenotypic characteristics of oxidative stress, increased expression of NADPH oxidase and IL-6, and increased cell proliferation and angiogenesis, which can be used to further study the physiopathological effect of 20-HETE in the cardiovascular system (Cheng et al., 2014).

## Conclusion

**FIGURE 2:**
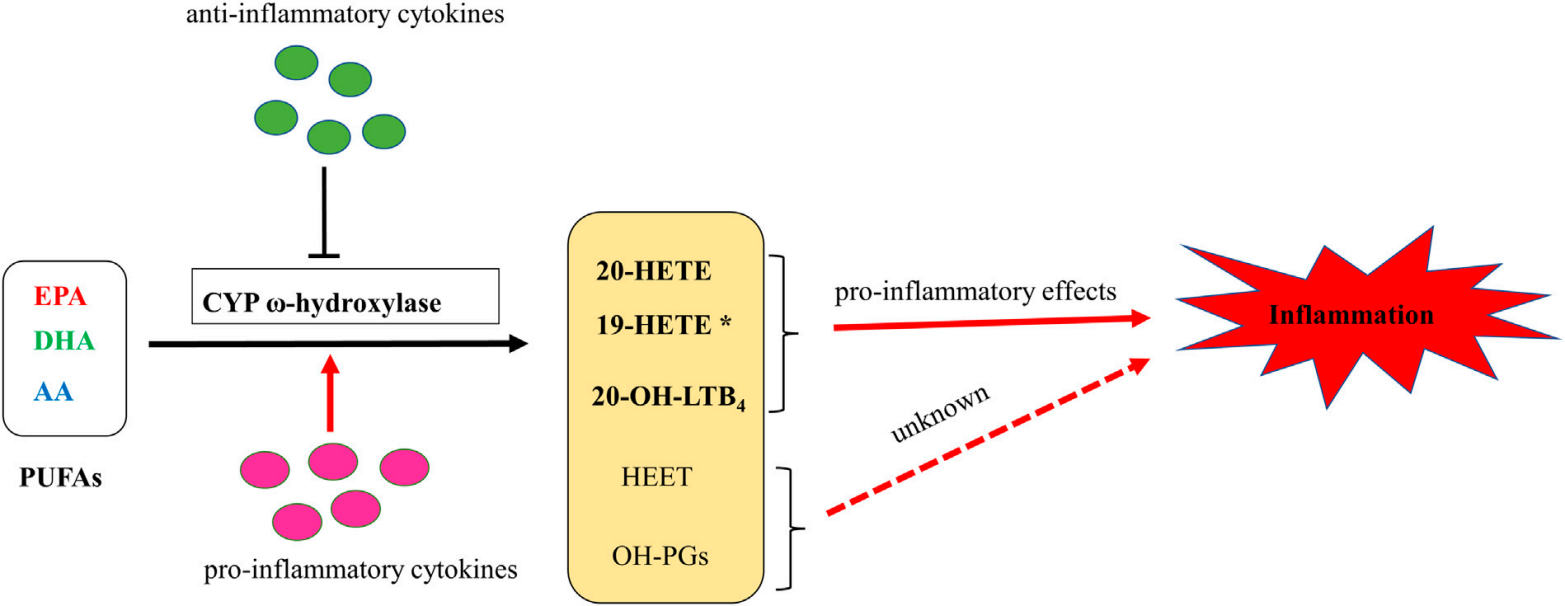
A putative schematic diagram of molecular mechanisms of CYP ω-hydroxylases-mediated eicosanoids on inflammation. * 19-HETE may take a pro-inflammatory role in chronic kidney disease. However, it may also act as an anti-inflammatory mediator since it antagonized 20-HETE-induced inflammation. In addition, 19(S)-HETE was reported to be more active than 19(R)-HETE against Ang II-cell hypertrophy.

**FIGURE 3:**
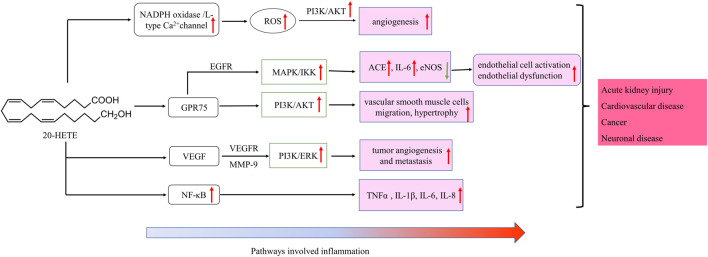
Schematic of signaling cascades involving 20-HETE in inflammation disease. ↑: up-regulation; ↓: down-regulation. IL, interleukin; LOX, MAPK/ERK, the mitogen-activated protein kinase/extracellular signal-regulated kinase; NF-κB, nuclear factor-kappa B; TNF-α, tumor necrosis factor alpha; EGFR, epidermal growth factor receptor, VEGF, vascular endothelial growth factor; ROS, reactive oxygen species; PI3K, phosphoinositide 3-Kinases.

CYP ω-hydroxylase and metabolite have been reported to play an important role in the inflammatory process (Figure 2). In a variety of inflammatory diseases, the activity of CYP ω-hydroxylase is regulated by inflammatory factors. Pro-inflammatory cytokines, IL-1β, IL-6 and TNF-α, can increase CYP ω-hydroxylase activity, whereas anti-inflammatory cytokines such as IL-10 can inhibit CTP hydroxylase expression. Therefore, the production of metabolites of these hydroxylases are affected accordingly. At present, a large number of studies showed that 20-HETE could modulate inflammatory processes (Figure 3). However, little is known about the role of other CYP hydroxylated products in inflammation. 20-HETE can increase the production of adhesion molecules and inflammatory cytokines as well as ROS level through the activation of NF-κB, MAPK pathway, and NADPH oxidase, to activate endothelial cell activation, promote cell proliferation and regulate endothelial dysfunction. The accumulation of inflammatory factors will also affect the activity of CYP ω-hydroxylases to promote the metabolism of eicosanoids and form a positive feedback regulation, further affecting the progress of cardiovascular diseases, cancer, inflammation and other diseases. Elucidation of the effects of inflammation and infection on the metabolism of CYP hydroxylase and eicosanoids and the relationship between specific cytokines and their mediated of CYP enzymes will help in-depth understanding about the pathogenesis of many diseases and update therapeutic strategies. However, due to the complexity of the cytokines involved in the inflammatory process and their signaling pathways, there has not been a consensus on its potential mechanism. Regulation of the expression or activity of CYP ω-hydroxylase may play a role in the treatment of inflammatory diseases. For the translational and clinical research of CYP-ω-hydroxylase, inducers and inhibitors of CYP-ω- hydroxylase may be novel therapeutic strategies for many clinical inflammatory diseases. In addition, CYP-ω-hydroxyase also could be used as the marker for the diagnosis of related difficult and complicated diseases, improving the existing diagnostic methods. Therefore, more researches are needed to further clarify the mechanism of CYP ω-hydroxylase to advance the translational and clinical studies of CYP ω-hydroxylases.

## Glossary

AA: arachidonic acid
ACE: angiotensin converting enzyme
COX: cyclooxygenase
CYP: cytochrome P450
DHA: docosahexaenoic acid
DHET: dihydroxyeicosatrienoic acid
DiHOME: dihydroxyoctadecenoic acid
EDPs: epoxydocosapentaenoic acid
EEQ: epoxyeicosatetreaenoic acid
EET: epoxyeicosatrienoic acid
EGFR: epidermal growth factor receptor
EPA: eicosapentaenoic acid
HDoHE: hydroxydocosahexaenoic acid
HEETs: hydroxyepoxyeicosatrienoic acids
HEPEs: hydroxyeicosapentaenoic acid
HETE: hydroxyeicosatetraenoic acid
HPDHA: hydroperoxydocosahexaenoic acid
HPEPE: hydroperoxy-eicosapentaenoic acid
IL: nterleukin
LOX: lipoxygenase
LPS: ipopolysaccharide
LTA4: eukotriene A4
LTB4: leukotriene B4
LXs: Lipoxin
MAPK/ERK: the mitogen-activated protein kinase/extracellular signal-regulated kinase
NF-α: tumor necrosis factor alpha,
NF-κB: nuclear factor-kappa B
PGE3: prostaglandin E3
PGH2: prostaglandin H2
PI3K: phosphoinositide 3-Kinases.
PLA2: phospholipase A2
PPAR: peroxisome proliferator-activated receptor
PUFA: polyunsaturated fatty acid
ROS: reactive oxygen species
sEH: soluble epoxide hydrolaseT
VEGF: vascular endothelial growth factor
